# Polymerase chain reaction‐positivity and predictors for SARS‐CoV‐2 infection among diagnosed cases' in North West Ethiopia

**DOI:** 10.1002/hsr2.1663

**Published:** 2023-10-26

**Authors:** Fassikaw Kebede, Tsehay Kebede

**Affiliations:** ^1^ Department of Epidemiology & Biostatics, College of Health Science Woldia University Woldia Ethiopia; ^2^ Department of Geography, Faculty of Social Science Bahirdare University Bahirdare Ethiopia

**Keywords:** COVID‐19, ethiopia, positivity, RT‐PCR

## Abstract

**Background:**

The lack of sophisticated diagnosing equipment for polymerase chain reaction (PCR) during the incidence of variant types of COVID‐19 underestimates the morbidity and mortality patterns of this pandemic. Thus, this study aimed to estimate seropositive and confirmatory predictors for COVID‐19 suspected and tested cases through polymerase chain reaction (RT‐PCR) in two diagnosing

**Methods:**

**A** facility‐based descriptive cross‐sectional study was employed among COVID‐19 suspected cases from January 2, 2022, to June 9, 2022. The data were collected both using a structured interviewees and nasopharyngeal (NP) swabs. The nasal swab (NS) was analyzed in the laboratory for RNA detection of the virus using PCR. The collected data were entered into Epi Data version 4.2 and then exported to STATA (SE) version R‐14 software for further analysis. multivariable logistic regression was used to assess the associated risk

**Results:**

A total of 285 suspected cases have participated in this study. The overall mean (±SD) age of the participants was 37.5 (±18.5) years. The majority, 174 (61.1%) of the tested groups were symptomatic when diagnosed. The positivity of RT‐PCR for suspected and COVID‐19 diagnosed cases were confirmed in 62/285 (21.75%). In multivariable analysis, they were aged 26–50 years (adjusted odds ratio [AOR] = 4.2, 95% confidence interval [CI] = 1.5–10.75), had comorbidity (AOR = 5.8; 95% CI = 2.1–12.2), and cigarette smokers (AOR = 13.5; 95% CI = 5.3–36.6) were significantly associated with developing COVID‐19 infection.

**Conclusion:**

More than two in every nine suspected cases were positive RT‐PCR tests, and the infectivity of COVID‐19 was significantly associated with age 25–50 years, comorbidities, and cigarette smoking. The deployment of high‐quality diagnostic kits like RT‐PCR is crucial for the early detection and risk stratification of suspected cases.

## 1 INTRODUCTION

The coronavirus disease 2019 (COVID‐19) pandemic still causes an ongoing global pandemic and public health emergency.^1^, ^2^ The spectrum of severity in COVID‐19 varies broadly, from asymptomatic infection to severe complications like organ failure to death.^3^ Epidemiologically, asymptomatic or silent spreaders have been one of the major causes of the rapid transmission of SARS‐CoV‐2 infections.^4^, ^5^, ^6^ The global community has been passing three epidemic surges of SARS‐CoV‐2 through causing different COVID‐19 variants.^7^ To date, there have been seven known strains of human coronaviruses (HCoVs) belonging to four genera (i.e., α‐, β‐, γ‐, and δ‐CoV) which have been recognized as HCoV‐NL63, HCoV‐229E, HCoV‐OC43; HCoV‐HKU, SARS‐CoV, MERS‐CoV; and SARS‐CoV‐2.^7^, ^8^ Recent evidence shows that the Omicron variant has a growing advantage over the delta, Gamma, and Beta variants with a doubling time of 2–3 days.^9^

In most COVID‐19 testing and diagnosing centers, test accuracy studies, reverse transcriptase polymerizing chain reaction (RT‐PCR) is defined as the gold standard, that is, having 100% diagnostic affinity of sensitivity and specificity for RNA detection of SARS‐CoV‐2 antigens.^1^, ^5^, ^9^ Detection methods based on nucleic acid amplification tests (NAAT) are popular and preferable for COVID‐19 in many developed and developing countries^10^ and depend upon the detection of viral genetic components of viral RNA.^1^ There are two peaks of kurtosis of the epidemic; the first wave was from August to September 2020, and the second wave occurred from February to March 2022.^2^ The overall new incidence of the new variant of Omicron variants was more than 50% rocketed in resource‐limited African countries including Ethiopia.^11^ In fact, out of 3,574,218 total suspected and tested cases since the first COVID‐19 case was reported, 493,000 cases were confirmed and 7571 deaths, as of August 10, 2022, were reported.^7^, ^8^ As reported by different research^11^, ^12^, ^13^ suspected COVID‐19 in fever, fatigue, dry cough, and global ache cases was zero missing on RT‐PCR tested with the correct sample specifications sampling. However, in most resources, the limited setting methods used for the test of COVID‐19 were substantially varied between settings^14^; which underestimates the actual morbidity and mortality pattern. Despite antigen methods of rapid diagnostic tests have been developed for large‐scale screening of populations, the power of sensitivity for COVID‐19 detections is only 22.9%–75.9% as compared with 100% affinity of RT‐PCR,^2^, ^15^ While high sensitivity and specificity are important not to miss the suspected cases beyond of minimizing false positivity.^6^ The spread of severe acute SARS‐CoV‐2 in Ethiopia is below the par understood and to date has been poorly characterized by a lower number of confirmed cases and deaths in other regions of the sub‐Sahara African (SSA).^11^, ^16^ Thus, this study is aimed to estimate the proportions of Positivity and confirmatory predictors for COVID‐19 suspected and diagnosed SARS‐CoV‐2 clinical Samples of 285 cases in two diagnosing centers from January 2, 2022, to June 9, 2022.

## 2 METHODS

### 2.1 Study area, study design, and setting

A hospital‐based descriptive cross‐sectional study was conducted from January 2, 2022, to June 9, 2022, in two centers of Pawe and Injibara comprehensive specialized hospitals. The Throat/Nasopharyngeal swabs (NS) were collected among 285 COVID‐19 eligible participants who fulfilled the WHO criteria for COVID‐19 suspected cases.^17^, ^18^

### 2.2 Suspected cases

Patients with an acute respiratory illness like (fever and at least one sign or symptom of respiratory disease, e.g., cough, shortness of breath), and a history of traveling abroad, report community transmission of COVID‐19 disease 2 weeks before symptom onset and/or a patient with any Acute respiratory illness and has been in contact with confirmed cases.^1^, ^2^

### 2.3 Sample size determination

The sample size of 285 was calculated using a single population proportion formula with the following descriptions *N* = (*Z*_*a*/2_)^2^ *P* (1‐*P*)/*d* ^2^. By considering this assumption (*Z* a1/2 = 0.05% with two‐sided = 1.96), the margin of error (*d* = 5%), seroprevalence from previous studies 4.7%,^11^ and adding a 5% nonresponse rate were found to be the final sample 247. However, from January 2, 2022, to June 9, 2022, there was only 287 cases were tested and reported. Therefore, since it is manageable, we used a survey rather than applying a sampling procedure.

### 2.4 Detection of viral SARS‐CoV‐2 by RT‐PCR

RT‐PCR Kit uses the latest developments in reverse transcriptase (RTase) technology and buffer chemistry for efficient cDNA synthesis in a single tube were used for detections of SARS‐COV‐2 viral RNA.^2^ Following the viral RNA extraction, the extraction sampled was was detected by a commercially available one of the BGI types of RT‐PCR assays with recommendations and instructions on a Quant Studio 5 DX real‐time PCR system (Thermo Fisher Scientific). Master Mix preparations were used to increase the sensitivity and specificity of RT‐PCR.^1^, ^3^, ^19^

### 2.5 Test kit selection

A reverse transcriptase‐PCR test has been designed based on targeting parts of SARS‐COV‐2: spike (S), open reading frame1a/b (ORF1a/b) b‐nuclear shuttle protein (ORF1b–nsp14), envelope (E), RNA‐dependent detection up one RNA polymerase (RdRp) from the structural part of viral antigen. Accordingly, the current study evaluated test kits that employed E gene, RdRp, and N SARS‐COV‐2 viral genes in Ethiopia which are commercially available.^2^, ^3^

Laboratory RNA purification; the specimen was drowned or collected from the suspected cases of specimens of nasopharyngeal (NS) with 2 mL VTM (China Miracle Technology Co. Ltd (www.mantacc.com) by a professionally trained laboratory technologist for RNA purification. Then briefly, 200 μL NP samples were transferred into a 1.5 mL Eppendorf tube. Following this, 50 μL of proteinase K and 200 μL of buffer were added after brief centrifugation and incubation of the tube at 72°C for 10 min. The cut‐off value for a positive test is ≤38 *Ct*, and any value ≥38 is regarded as a negative test. The PCR program consisted of 50°C for 20 min, 95°C for 10 min followed by 40 cycles of 95°C for 15 s and 60°C for the 30 s.^1^, ^6^, ^11^ The remaining procedure was performed as per the RNA/DNA purification kit insert.^20^, ^21^ The amplification of the NA was run based on Bio‐Rad CFX96 Touch Real‐Time PCR.

### 2.6 Outcome ascertainment

Detection of SARS‐CoV‐2 from suspected cases or a patient was collected through nasopharyngeal (NS) swabs by a trained clinical laboratory technologist, and samples were tested by RT‐PCR assay irrespective https://www.uptodate.com/contents/covid-19-diagnosis and https://www.cdc.gov/coronavirus/2019-ncov/lab/guidelines-clinical‐ of clinical symptoms for cases when suspected and having contact with COVID‐19 infection.^1^, ^22^ The outcome of the end RT‐PCR assay test was declared in the laboratory registration log book with positive/negative definitions. Which is already formatted by the Federal ministry of health^1^ and includes the following predefined variables such as sex, age, residence, marital status, level of education, religion, and status (mask‐wearing, alcohol drinking, smoking, quarantining) were incorporated for the determination of PCR positivity rate.^1^, ^11^

### 2.7 Data quality assurance

All procedures and steps were pretested and proper amendments were taken before the actual data collection. The quality was ensured through standardized data collection tools adapted from the registration book of COVID‐19 diagnosing and reporting format^1^ and from previously published literature.^2^, ^10^, ^15^, ^16^ Two laboratory professionals who trained for sample collection for all COVID‐19 cases and a supervisor for overall data collection activities were trained for 2 days. A 5% pretest was done in suspected cases before the actual data collection was been to check the clarity of the checklists and the availability of listed variables existence in the results reporting format.

### 2.8 Data management and analysis

After editing and coding, the collected data were entered into Epi Data version 4.2, and then exported to STATA (SE)/14 for further analysis. Before analysis, the data were cleaned, and simple frequency, cross‐tabulation, and categorization of continuous variables were done to be suitable for analysis. A logistics regression was fitted to determine factors contributing to the risk of COVID‐19 infection during diagnosis through RT‐PCR. The assumption of the logistics regression model was checked by the chi‐square test. Categorical variables with a *p* < 0.25 in the bi‐variable analysis were candidate transferees for multivariable logistics regression analysis. The strength of association between RT‐PCR positivity and confirmatory predictors for COVID‐19 cases was expressed as an adjusted odds ratio (AOR) with 95% confidence interval (CI), at *p* < 0.05.

## 3 RESULT

### 3.1 Socio‐demographic characteristics

Overall, 287 participants were recruited; information was collected from 285 making the overall response rate 99.3%. The majority 223 (61.26%) of the participants were male in gender, and 233 (60.5%) were urban inhabitants. The overall mean (±SD) age of participant children was found to be 32.3 (±15.4) years. We have three main sources of diagnosed populations, of which nearly half of the participant cases 169 (46.43%) were from suspected groups, followed by the community (Road) surveillance 118 (32.42), and the remaining were from index case contact history 77 (21.15%) as indicated in Table 1.

**Table 1:** Baseline socio‐demographic characteristics of suspected and diagnosed cases.

Variables	Categories	Frequency	Percent
Age (Years)	≤25 years	94	32.9
	26–50 years	145	50.8
	≥50 years	46	16.5
Sex	Male	179	62.81
	Female	106	37.2
Resident	Urbane	170	59.6
	Rural	115	35
Marital status	Married	164	59.6
	Divorced	79	26.6
	Never in union	42	13.6
Occupation	Employer	45	15.79
	Farmer	79	27.7
	Merchant	76	26.6
	Student	39	13.6
	Merchant	46	16.2
Education	Unable to read and write	56	19.13
	Complete high school	93	32.4
	Complete preparatory	84	29.3
	Diploma and above	52	25.2
Religion	Orthodox	76	26.6
	Muslim	81	28.4
	Protestant	63	22.1
	Catholic	65	22.2
Alcohol drinking status	Yes	70	75.4
	No	215	24.6
Smoking status	Yes	59	20.7
	No	226	79.3
Comorbidity	Yes	78	27.3
	No	207	72.6
Daily use of face mask	Yes	82	28.3
	No	203	71.6
History of quarantined	Yes	55	19.3
	No	230	80.9
Absenting from crowded area	Yes	106	37.2
	No	179	62.8
Daily uses of hand sanitizer	Yes	74	25.5
	No	211	74.4
Staying at home during a pandemic	Yes	119	41.7
	No	166	58.2
Social distance	Yes	**127**	44.5
	No	158	55.4
Reason for testing COVID‐19	Symptomatic (ST)	174	60.1
	High‐risk groups (HRG)	52	18.2
	History of contact (HC)	28	9.8
	Repeated test (RT)	18	6.34
	Confirmatory test (CT)	13	4.6
Clinical decision tested	Isolation	41	14.5
	Home quarantined	179	62.8
	Hospitalized	42	14.5
	Confirmatory	18	6.32
	Others	5	1.75

### 3.2 Presenting symptoms of suspected cases

**Figure 1:**
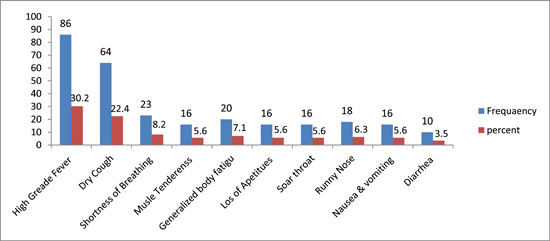
Presenting signs and symptoms of suspected cases during diagnosis in the two centers.

Regarding the presenting symptoms of suspected cases before COVID‐19 was diagnosed that were reported among the patients; almost all patients had the clinical features of fever (30.2%) followed by coughing 22.4%, difficulty of breathing 8.3%, generalized body fatuge 7.2%, muscle tenderness 5.6%, loss of appetites 5.6%, sore throat 5.6%, runny nose 6.3%, nausea and vomiting 5.6%, and the remaining 3.5% were diarrhea, respectively as shown in Figure 1.

### 3.3 Prevalence of RT‐PCR‐diagnosed cases

**Figure 2:**
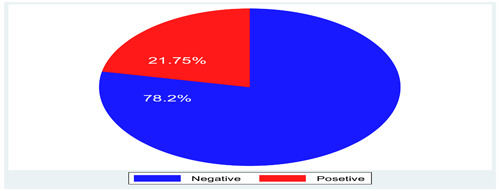
COVID‐19 confirmed cases thought RT‐PCR tested in two centers, North West Ethiopia.

Among the total of 287 COVID‐19 suspected and diagnosed cases, 285 individuals were tested for RT‐PCR and authorized their results. They are diagnosed with different risks of exposure. Accordingly, the majority 174 (61.1%) of them were due to symptomatic, whereas 52 (18.25%) were high‐risk group, 28 (9.82%) had contact history with cases, checkup test 18 (6.32%), and confirmatory test 13 (4.56%). Accordingly, 21.75% (95% CI = 17.3%–26.9%) of suspected participants were confirmed by the RT‐PCR test (Figure 2).

### 3.4 Confirmatory predictors for risk groups

A statistically significant difference among COVID‐19 suspected and RT‐PCR diagnosed cases in different groups of variables of alcohol drinking, sex, and co‐morbid illness. Based on this, a significantly higher risk difference was observed for urban and rural residents, meaning the urban dweller has a significantly higher proportion of acquiring of COVID‐19 infection than rural inhabitants 45 (72.5%) versus 17 (27.4%) with *χ*² *df* = 1; 9.6, *p* = 0.02) as shown in Table 2. Likewise, suspected cases having alcohol drinking habits had a greater risk of COVID‐19 confirmed diagnosis as compared with the non‐alcohol drinker group as stated here (61.2% vs. 38.7%) with *χ*² *df* = 1; 70.1, Pr = 0.001). There were also significant positivity risk differences for COVID‐19 among cases attending social media during the COVID‐19 pandemic with user group 16 (17.2%) versus nonuser group 46 (74.2%) with evidence *χ*² *df* = 1; 15.2, Pr = 0.001) as shown in Table 2.

**Table 2:** Socio‐demographic characteristics of COVID‐19 suspected and diagnosed participants in two centers.

Variables	Categories	Frequency	Percent	*χ* ^2^	*p*‐Value
Resident	Rural	115	40.5	9.2	0.002
	Urban	170	59.6		
	Married	164	59.6	2.29	0.21
Marital status	Divorced	79	26.6		
	Never in union	42	13.6		
	Employer	45	15.7		
Occupation	Farmer	79	27.7	8.2	0.07
	Merchant	76	26.6		
	Student	39	13.6		
	Merchant	46	16.2		
Use of social media	Yes	136	47.1	22.6	0.001
	No	149	52.9		
Alcohol drinking status	Yes	70	75.4		
	No	215	24.6	23.6	0.001
Smoking status	Yes	59	20.7		
	No	226	79.3	70.5	0.001
Comorbidity	Yes	59	20.3		
	No	226	79.6	218.6	0.001
Daily use of face mask	Yes	82	28.3		
	No	203	71.6	1.8	0.21
History of contact	Yes	55	19.3	3.28	0.05
	No	230	80.9		
Absenting from crowded area	Yes	106	37.2	1.8	0.19
	No	179	62.8		
Daily uses of hand sanitizer	Yes	83	28.5	5.7	0.002
	No	202	77.4		
Staying at home during a pandemic	Yes	119	41.7		
	No	166	58.2	4.76	0.03
Social distance	Yes	**127**	44.5		
	No	**158**	55.5	5.86	0.02
RT‐PCR result	Positive	62	21.8		
	Negative	223	78.1		

### 3.5 Confirmatory predictors for COVID‐19 suspected and diagnosed cases

**Figure 3:**
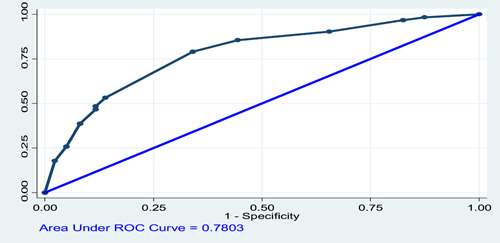
Model goodness of fit test. Number of observations = 285. Number of groups = 10. Hosmer–Lemeshow test = 0.975. Prob *>* (*χ*²) = 0.946.

After controlling for certain confounding, multivariable logistic regression, variables like age, smoking habit, and baseline comorbidities have been associated with the acquisition of COVID‐19 infection. After controlling a certain founding, the model was adequate on 8 variables with three significant predictors for COVID‐19 infection as shown in rock curve (Figure 3).

Accordingly, compared with patients aged younger than 25 years, the suspected cases aged 26–50 years were nearly four times more likely to develop COVID‐19 (AOR = 4.2, 95% CI = 1.5–10.75). Likewise, compared to non‐smokers of a cigarette, being a cigarette smoker is nearly 14 times increased the likelihood of acquiring COVID‐19 infection (AOR = 13.5; 95% CI = 5.3–36.6) Moreover, the likelihood of developing COVID‐19 infection for suspected cases with comorbidity was nearly six times increased as compared with counter groups (AOR = 5.8; 95% CI = 2.1–12.2) (Table 3).

**Table 3:** Bi‐variable and multivariable logistic regression for confirmatory predictors' of COVID−19 suspected and diagnosed cases in two centers.

Variables	Categories	RT‐PCR tests		95% confidence interval (CI) CHR	95% CI (AHR)	*p* ‐Value
		Polymerase chain reaction (PCR) positive *N*	PCR negative *N*			
Age	≤25 years	13 (5.5%)	79 (27.7%)	Ref	Ref	
	26‐50 years	38 (13.3%)	104 (36.4%)	2.2 (1.18–4.4)	4.2 (1.5–10.7)	0.01*
	≥50 years	11 (3.8%)	40 (14.1%)	1.6 (0.68–4.2)	1.8 (0.54–5.95)	0.35
Sex	Male	37 (12.9%)	142 (49.8%)	Ref	Ref	0.52
	Female	25 (8.7%)	81 (28.4%)	1.2 (0.7–2.1)	1.3 (0.55–2.7)	
Resident	Urbane	45 (15.7%)	125 (43.8%)	2.1 (1.12–3.8)	3.3 (0.3–7.4	0.20
	Rural	17 (5.9%)	98 (34.3%)	Ref	Ref	
Marital status	Married	28 (9.8%)	133 (46.6%)	Ref	Ref	
	Unmarried	34 (11.9%)	90 (31.5%)	1.5 (1.2–3.2)	1.56 (0.9–2.7)	0.11
Alcohol drinking	Yes	34 (11.9)	60 (21.1%)	3.3 (1.89–5.8)	1.4 (1.12–3.5)	0.12
	No	28 (9.8%)	163 (57.2%)	Ref	Ref	
Cigarette smoking	Yes	33 (11.57%)	31 (10.8%)	7.2 (3.7–13.3)	13.5 (5.3–36.6)	0.01*
	No	29 (10.2%)	192 (67.3%)	Ref	Ref	
History of contact	Yes	33 (11.6%)	41 (14.3%)	1.2 (0.7–9.2)	1.2 (0.9–2.5)	0.23
	No	29 (10.2%)	182 (63.8%)	Ref	Ref	
Social media usage	Yes	16 (5.5%)	120 (42.1%)	3.3 (1.78–6.3)	1.5 (0.9–6.6)	0.31
	No	46 (16.6%)	103 (36.1%)	Ref	Ref	
Stayed at home	Yes	23 (8.1%)	105 (36.8%)	Ref	Ref	
	No	39 (13.6%)	118 (41.4%)	1.7 (0.84–2.6)	1.4 (0.75–2.46)	0.49
Comorbidity	No	23 (8.1%)	206 (72.2%)	Ref	Ref	
	Yes	38 (13.3%)	17 (5.9%)	15.2 (7.7–25)	5.8 (2.1–12.2)	0.02*

* Statistical significant of variables.

## 4 DISCUSSION

At the end of the study period, the overall positivity of RT‐PCR among COVID‐19 suspected and diagnosed cases were confirmed in 62/285 (21.75%) with (95% CI = 17.3–26.9). Which is inconsistent with previous finding in 3.5% in Addis Ababa,^5^ 3.2% in Dire Dawa,^3^ 2.7% in Turkey,^23^ 2.5% California,^24^ and 8.3% in Israel.^25^ The possible reason for the variation of this finding can be due to the differences in the study settings, diagnostic ability, and the significant variation in study periodic where the pandemic wave urges high. Conversely, the estimated seroprevalence of SARS‐CoV‐2 consistent with 13.0% in Mexico,^26^ 15.4% reported in France,^27^ and 20.78% reported in India.^28^ This might be due to the similarity of the stages of infectivity and the difference is the presence of enforcement of rules on populations' restrictions for the COVID‐19 pandemic. Regarding predictors for COVID‐19 infections, the suspected cases being aged 26–50 years were nearly four times increased likely hood acquiring COVID‐19 infection, compared with aged ≤25 years (AOR = 4.2, 95% CI = 1.5–10.75). This is consistent with previous findings in Pawe hospital,^11^ reported in two hospitals,^29^ and found in urban areas, in central Ethiopia,^14^ and in Italia.^30^, ^31^ Consistent findings reported in Nigeria,^32^ Iran,^33^, ^34^ and Italy,^30^ baseline comorbidity increased the likely hood of risk for infection of COVID‐19. Accordingly, baseline comorbidity increased six times the infectivity of COVID‐19 when tested through RT‐PCR as compared with no comorbidity groups (AOR = 5.8; 95% CI = 2.1–12.2).

Comorbidity is significantly associated with risk for poorer clinical outcomes for infected cases in SARS‐CoV‐2. This is similar with our reports; more half of 70.1% participants had one or more than comorbidities. This is consistent earlier reported in Addis Ababa,^35^ and Nekemte^36^ hospitals; the risks of getting COVID‐19 infection was double fold increased, and associated with delayed in viral‐clearance an prolong transmission periods.^35^, ^36^, ^37^ In this study, compared with cigarette nonsmoker group, COVID‐19 suspected and tested smoker groups increased the likely hood of COVID‐19 infection 14 times (AOR = 13.5; 95% CI = 5.3–36.6). Which is consistent with the previous report in Malaysia^38^ and confirmed that tobacco is a known risk factor for severe disease in many respiratory infections, including coronaviruses, SARS‐CoV‐2 further impairs the immune system and causes for poor prognosis.

### 4.1 Strength and limitations of the study

To the best of our knowledge, this is the first laboratory‐based PCR study among COVID‐19 suspected individuals from Ethiopia, which includes clinical symptoms and risk factors for infection conducted by a multidisciplinary research team. Where as the sample size was relatively too small. Additionally, since this study was conducted on blood parameters by RT‐PCR, not every patient was continuously monitored for all clinical manifestations including serum and clinical chemistry of suspected cases.

## 5 CONCLUSION

More than two in every nine suspected cases were positive RT‐PCR tests, and the infectivity of COVID‐19 was significantly associated with age 25–50 years, comorbidities, and cigarette smoking. The deployment of high‐quality diagnostic kits like RT‐PCR is crucial for the early detection and risk stratification of suspected cases.

## AUTHOR CONTRIBUTIONS

**Fassikaw Kebede**: Conceptualization; data curation; formal analysis; funding acquisition; investigation; methodology; project administration; resources; software; supervision; validation; visualization. **Tsehay Kebede**: Conceptualization; data curation; formal analysis; funding acquisition; investigation; methodology; project administration; resources; software; supervision; validation; visualization; writing—original draft; writing—review and editing.

## CONFLICT OF INTEREST STATEMENT

The authors declare no conflict of interest.

## ETHICS STATEMENT

All methods were performed following the relevant guidelines and regulations. The Ethical Review Board of Woldia University Ethically cleared with Refill No WU/CHS/2013/117. Written informed consent was obtained from every participating case after the purpose risk; benefits, confidentiality, and degree of involvement were fully explained to caregivers in their local language before starting the data collection. For participants less than 18 years of age, writing was obtained from caregivers.

## TRANSPARENCY STATEMENT

The lead author Fassikaw Kebede affirms that this manuscript is an honest, accurate, and transparent account of the study being reported; that no important aspects of the study have been omitted; and that any discrepancies from the study as planned (and, if relevant, registered) have been explained.

## Data Availability

The datasets employed in this study are available with the corresponding author upon reasonable request via email.
